# CARD15/NOD2, CD14 and Toll-like 4 Receptor Gene Polymorphisms in Saudi Patients with Crohn’s Disease

**DOI:** 10.3390/ijms13044268

**Published:** 2012-04-02

**Authors:** Nahla Azzam, Howaida Nounou, Othman Alharbi, Abedulrahman Aljebreen, Manal Shalaby

**Affiliations:** 1 Department of Internal Medicine, Division of Gastroenterology, Faculty of Medicine, King Saud University, Riyadh 11461, Saudi Arabia; E-Mails: nahla5_99@yahoo.com (N.A.); alharbiothman@hotmail.com (O.A.); amaljebreen@gmail.com (A.A.); 2 Department of Biochemistry, Faculty of Medicine, Alexandria University, Alexandria 21111, Egypt; 3 Department of Biochemistry, Faculty of Science, King Saud University, Riyadh 11495, Saudi Arabia; E-Mail: mashalaby@ksu.edu.sa

**Keywords:** inflammatory bowel disease, Crohn’s disease, genetic polymorphism, *CARD15/NOD2*

## Abstract

Crohn’s disease (CD) is a multifactorial disease with a genetic component and an observed association with genes related to the innate immune response. Polymorphisms in the *CARD15/NOD2* gene, in addition to functional variants of the toll-like receptor-4 (TLR4) and CD14 genes, have been associated with the development of Crohn’s disease. There is no information about the frequency of these polymorphisms in the Saudi population. We examined the frequency of the three major *CARD15/NOD2* risk alleles (Leu1007fsinsC, Arg702Trp, and Gly908Arg*)* and the TLR4 (Thr399Il) polymorphism as well as a functional polymorphism in the promoter of the CD14–159C/T in 46 Saudi CD patients and 50 matched controls. Genotyping was performed by allele-specific PCR or by restriction fragment length polymorphism (PCR-RFLP) analysis. The mutant genotype frequencies of the Leu1007fsinsC, Arg702Trp and Gly908Arg in the patient group were 6.5, 21.7 and 6.5%, respectively, compared with frequencies of 0, 4 and 2%, respectively, in the control group. There were 15 patients who carried the mutant alleles for all three *CARD15/NOD2* variants, Leu1007fsinsC, Arg702Trp and Gly908Arg, while none of the control candidates carried the three alleles. This genetic study provides evidence that the three major *CARD15/NOD2* variant alleles and the CD14 –159C/T polymorphism are associated with Crohn’s disease (CD) susceptibility in the Saudi population; however, there is no evidence that the TLR4 (Thr399Il) or *CARD15/NOD2* polymorphisms can be considered risk factors for Crohn’s disease.

## 1. Introduction

Crohn’s disease is a chronic, nonspecific, idiopathic gastrointestinal inflammatory disease that often leads to fibrosis and obstructive symptoms and can affect any part of the gastrointestinal tract from the mouth to the anus. The etiology of Crohn’s disease has not been clearly identified. Many factors have been suggested, but none are proven. Possible risk factors include immunologic factors, infectious agents (such as bacteria, viruses or amoebae), and dietary factors (including chemicals and drugs) [1].

Over the last decade, major advances have occurred that contribute to the understanding of the genetics of Crohn’s disease (CD), including studies based on single nucleotide polymorphism (SNP) and candidate gene approaches and studies in mouse experimental colitis using transgenic and deletion (knockout) techniques. Genome-wide linkage screens have identified more than 31 genetic loci containing putative inflammatory bowel disease (IBD) risk factors that could be associated with CD; however, estimates suggest that these 31 loci only explain approximately 20% of the heritability of Crohn’s disease [2].

The discovery of *CARD15/NOD2* has focused interest on the regulation of innate immune responses and apoptosis. This gene, with important polymorphisms, encodes the intracellular bacterial sensor. The *CARD15/NOD2* gene is located on chromosome 16 and is expressed mainly in monocytes, dendritic cells, intestinal epithelium and Paneth cells [3]. The *CARD15/NOD2* protein recognizes components of the bacterial cell wall [4], is involved in the innate immune response and leads to the mobilization of the intracellular nuclear factor κB (NF-κB) [5].

The three major variants of *CARD15/NOD2*, Gly908Arg, Arg702Trp, and Leu1007fsinsC, are associated with a deficit in NF-κB activation in response to bacterial components, providing a unifying mechanism for the major CD-associated *CARD15/NOD2* variants [6]. Lesage *et al.* suggested that patients carrying two variant copies of the *CARD15/NOD2* gene are at increased risk of early age and fibrostenotic behavior of CD [2].

The contribution of the *CARD15/NOD2* gene to CD has been studied in different ethnic populations with positive associations in up to 50% of CD patients [2]. However, data from Asian countries such as Japan and China failed to show any significant association, and no data from the Arab world could support or deny such association [7,8]. Although it remains uncertain how or whether the *CARD15/NOD2* protein interacts with other protein families involved in the innate immune response, the *C*-terminal domain of *CARD15/NOD2* comprises a leucine-rich repeat (LRR) region, which has sequence homology with a number of plant disease resistance genes in the toll and toll-like receptor (TLR) gene superfamilies.

Toll-like receptor 4 (TLR4) was found to be strongly up-regulated in patients with CD, and the allele *299Gly* has been associated with the risk of developing IBD and particularly CD [9–11]. The TLR4 gene is expressed mainly in macrophages, dendritic cells and endothelial cells and to a lesser extent in the intestinal epithelium. The TLR family is involved in the recognition of pathogen-associated molecular patterns by the immune system. The TLR4 protein recognizes lipopolysaccharide (LPS) from gram-negative bacteria and binds to LPS using the extracellular LRR domain, which leads to intracellular activation of NF-κB [12].

Klein *et al.* demonstrated an association of CD with a functionally relevant single nucleotide polymorphism in the promoter of the CD14 gene (T/C at position −159) and suggested that the interaction of the *CARD15/NOD2* and CD14 polymorphisms increases the risk for developing CD [13]. The human CD14 gene lies on the long arm of chromosome 5 (5q31.1), a region (IBD5) that has been reported to be associated with CD [14]. Membrane-bound CD14 is expressed on monocytes and macrophages [15].

To date, there has been no study performed in Asian Arabia, in particular Saudi Arabia, to evaluate genetic risk factors for CD among the Arab ethnic populations despite the increasing prevalence of the disease. Therefore, we have investigated the contribution of the three common *CARD15/NOD2* mutations, as well as TLR4 and the CD14 −159C/T gene promoter polymorphism, to the predisposition to CD among the Saudi population.

## 2. Results and Discussion

The demographic and clinical characteristics of the CD patients are shown in Table 1. The demographic characteristics of the control population were similar to those of the patients. Among the CD patients, 31 males and 15 females, the mean age was 30.43 ± 10.20; 50% had a history of surgery; and 66% had been treated with biological therapy, 28 (62%) with infliximab and 3 (6%) with Adalimumab.

### 2.1. Frequency of *CARD15/NOD2* Polymorphisms

The genotype and allele frequencies of the three major *CARD15/NOD2* polymorphisms in 46 CD patients and 50 healthy controls are shown in Table 2. The mutant genotype frequencies of Leu1007fsinsC, Arg702Trp and Gly908Arg in the patient group were 6.5, 21.7 and 6.5%, respectively, compared with frequencies of 0, 4 and 2%, respectively, in the control group. The mutant allele frequencies of Leu1007fsinsC, Arg702Trp and Gly908Arg in the patient group were 78.3, 60.6 and 82.6%, respectively, compared with frequencies of 20, 26 and 28%, respectively, in the control group. The corresponding *P* values and Odd ratio (OR) and 95% confidence interval {95% CI} were as follows: *P* < 0.012, OR 5.29 (CI 2.71–10.29); *P* < 0.0001, OR 21.87 (CI 3.99–119.9) and *P* < 0.015, OR 21.0 (CI 1.81–243.2), respectively. The *P* values and OR {95% CI} for the mutant alleles were as follows: *P* < 0.0001, OR 7.71 (CI 2.87–20.71); *P* < 0.0001, OR 9.42 (CI 3.43–25.9) and *P* < 0.0001, OR19.0 (CI 6.20–58.21).

The mutant genotype for Leu1007fsinsC was not detected in any of the controls. Only one control subject carried the mutant genotype for both the Arg702Trp and Gly908Arg polymorphisms of *CARD15/NOD2*. There were 15 patients who carried the mutant alleles for all three *CARD15/NOD2* variants, Leu1007fsinsC, Arg702Trp and Gly908Arg, while none of the controls carried the three alleles.

### 2.2. Frequency of TLR4 Thr399Ile and the −159 (C/T) Polymorphism of the *CD14* Gene

Allele and genotype frequencies of TLR4 Thr399Ile and the CD14 −159C/T gene polymorphism are presented in Table 3. There were marginal differences in the frequencies for TLR4 Thr399Ile between CD patients and controls. The genotype mutant frequencies for TLR4 Thr399Ile were 8.7% for the patients and 8% for the controls (*P* < 0.495), while the allele mutant frequencies for TLR4 Thr399Ile were 39% for the patients and 28% for the controls (*P* < 0.226).

The CD14 −159C/T gene polymorphism is significantly associated with CD. The genotype mutant frequencies of the CD14 −159C/T were 15% for the CD patients and 10% for the controls (*P* < 0.075), while the mutant allele frequencies of the CD14 −159C/T were 26% and 15% (*P* < 0.002), respectively.

Table 4 shows the comparisons for different allele frequencies. The statistical analysis identified a significant difference between Leu1007fsinsC and TLR4 Thr399Ile (*P* < 0.001), Arg702Trp and TLR4 Thr399Ile (*P* < 0.002) and Arg702Trp with CD14 −159C/T (*P* < 0.001).

Positive correlations have been determined between Arg702Trp and Gly908Arg, with a correlation coefficient of *rs* = −356 (*P* = 0.008), and between TLR4 Thr399Ile and CD14 −159C/T, with a correlation coefficient of *rs* = −355 (*P* = 0.008).

### 2.3. Identification of the Most Effective Mutation among CARD15/NOD2 (Leu1007fsinsC, Arg702Trp, Gly908Arg), TLR4 Thr399Ile and CD14 −159C/T

Comparing the significance of each single mutation against the other mutations studied showed that the *CARD15/NOD2* Leu1007fsinsC was more effective than TLR4 Thr399Ile, and the difference was highly significant (*P* < 0.001); additionally, *CARD15/NOD2* Arg702Trp was more effective than the TLR4 Thr399Ile, and the difference was highly significant (*P* = 0.002). The *CARD15/NOD2* Gly908Arg was more effective than TLR4 Thr399Ile and the CD14 −159C/T mutation with significant differences of *P* = 0.025 and *P* < 0.001, respectively.

### 2.4. Comparisons between the Two Groups (Control and Patients) According to Combinations of Mutations

Analysis of the coexistence of the TLR4, *CARD15/NOD2* and CD14 mutated alleles showed a higher frequency in patients (30.4%) than in controls (8%) (Table 5).

### 2.5. Analysis of the Crohn’s Disease Susceptibility Haplotype on *CARD15/NOD2*

Haplotype frequencies for the Leu1007fsinsC, Arg908Trp and Gly908Arg alleles were estimated for controls and patients (Table 6). A score for each haplotype (Hap-score) was calculated and *P*-value was obtained for the significance of each Hap-score. The haplotype 1-2-1, which only differed from the protective 1-1-1 haplotype at Arg908Trp, was significantly associated with CD (OR 4.538; *P* < 0.0001). All haplotypes containing more than one mutation were significantly associated with disease.

### 2.6. Genotype–Phenotype Analysis of *CARD15/NOD2*: Univariate Analysis

There was no association between the three variants of *CARD15/NOD2* and age at diagnosis, sex, smoking, anatomical distribution of disease, or disease behavior at diagnosis. For more information, see Supplementary File.

## 3. Experimental Section

Blood samples from 46 patients with CD and 50 age- and sex-matched healthy individuals were collected at the IBD Outpatient Clinic at King Khalid University Hospital (KKUH) between May 2010 and February 2011. The diagnosis of CD was based on standard clinical, endoscopic, radiological, and histological criteria [1]. Clinical and demographic characteristics were recorded, including age at diagnosis, gender, family history, smoking habits, disease behavior, disease location, and need for surgery.

### 3.1. Ethical Approval

This study was conducted after review and approval of the Institutional Review Board of the Ethics Committee at KKUH, and each patient gave written informed consent.

### 3.2. Methods

Allele-specific polymerase chain reactions (PCRs) were used to detect TLR-4, Thr399Ile and *CARD15/NOD2* polymorphisms in DNA samples extracted from 5 ml of total blood samples, All PCR assays were performed in a 50 μL volume reaction containing 10 mmol/L Tris-HCl, pH 8.3, 50 mmol/L KCl, 2 mmol/L MgCl_2_, 250 μmol/L dNTPs, 0.20 μmol/L concentration of each primer, 200 ng of genomic DNA and 2.5 U of Taq DNA polymerase (Promega). All cycling conditions initiated with denaturation at 94 °C for 4 min followed by 35 cycles of denaturation, annealing and extension which varied according to primers as shown in Table 7, all PCR reactions were followed by terminal extension step at 72 °C for 5 min finally the reactions were then held at 4 °C until analysis. PCR products were electrophoresed in an agarose gel and visualized by ethidium bromide staining.

## 4. Conclusions

This study is the first to determine the prevalence of *CARD15/NOD2*, TLR4 and CD14 −159C/T gene mutations in Saudi patients with CD. This genetic study provides evidence that the three major *CARD15/NOD2* variant alleles and the CD14 −159C/T polymorphism are associated with Crohn’s disease susceptibility in the Saudi; however, there is no evidence that the TLR4 (Thr399Ile) polymorphism is associated with Saudi CD.

Comparison of the frequencies of the mutations (Table 4) shows that TLR4 Thr399Ile is the least significant contributor to CD disease compared with the other mutations. The three main *CARD15/NOD2* mutations are well-known CD-susceptibility alleles, but the risk associated with the mutations at the individual and population levels remains largely indeterminate. The data presented here and the reports from the literature [16–19] demonstrate that in different populations, large variations are observed for the frequencies of the common mutations. In Europe, *CARD15/NOD2* mutations (especially the Gly908Arg and Leu1007fsinsC mutations) are relatively rare (2.4–7%) in northern countries including Scotland, Finland, Norway, and Sweden. The frequencies of the mutations, especially the Leu1007fsinsC mutation, are relatively high (7–11.5%) in Central Europe including Ireland, United Kingdom, Belgium, France, Germany, Croatia, and Hungary. Finally, in Southern countries, including Spain, Portugal, and Italy, the mutation frequency is intermediate (6–7.5%) with a lower frequency for the Leu1007fsinsC mutation [20]. In our study, the frequencies of the *CARD15/NOD2* mutations in the control group were 0–4% (Table 2), with the lowest frequency for the Leu1007fsinsC mutation.

All these data (with the exception of those for Greece, where a very high rate of Leu1007fsinsC and a very low rate of R702W mutations are observed) are in accord with the more general observations of North-South gradients for genetic polymorphisms in Europe that are compatible with a spread of Neolithic farmers from the Levant 10,000 years ago [20]. The low penetrance observed in at-risk genotypes demonstrates that *CARD15/NOD2* does not delineate a subgroup of simple Mendelian diseases.

Our data confirmed that *CARD15/NOD2* mutations are more common in CD patients than in a the control Saudi population, although our data showed that these mutations were not the only CD risk factor and that additional genetic and/or environmental cofactors not discovered yet are required for disease development at the individual and population levels.

The polymorphism in the CD14 gene has been shown to increase TNF-α production in response to bacterial LPS and to amplify mucosal immune responses, suggesting that CD14 may play a role in the etiology of CD. Previous studies showed that the polymorphism in the promoter of the human CD14 gene was associated with CD and ulcerative colitis (UC), either alone or through interaction with polymorphisms in the *CARD15/NOD2* gene [21]. We have shown that the CD14 −159C/T polymorphism in the CD14 gene was more common in CD patients than in a control Saudi population; however, an association of the CD14 −159C/T polymorphism with CD in other ethnic populations was heterogeneous and scanty.

The importance of TLR4 polymorphisms in CD is less clear than the importance of the *CARD15/NOD2* mutations. Franchimont *et al.* have reported a two-fold elevation in allele frequency of TLR4 Asp299Gly in Belgian CD patients [22]. The TL4 Thr399Ile variant was associated with UC in a German study [23]; however, Japanese [24] and Hungarian studies [25] failed to detect any individuals displaying the mutant alleles of TLR4.

The three candidate genes of CD14, *CARD15/NOD2* and TLR4, studied in a Tunisian population [26], were not associated with CD, contrary to the results obtained with European and American populations [10].

These findings support our results because in our study of TLR4 polymorphisms no significant differences in the allele or genotype frequencies between the patient and control groups were identified. The small size of our samples may have contributed to these negative results.

The coexistence of the TLR4 and CD14 mutated alleles was higher in CD patients than in healthy subjects, and this association with increased risk of CD in the Saudi population was significant.

Analyzing the SNP interactions will contribute to an understanding of the phenotypic variation of the disease, but currently, the results with CD are controversial. In fact, some studies show that the coexistence of mutations of the *CARD15/NOD2* and TLR4 genes increases the risk of developing CD [27,28] or is associated with the phenotype of the disease, but these results have not been confirmed.

The coexistence of the TLR4, *CARD15/NOD2* and CD14 mutated alleles was not the same in CD patients and controls (Table 5). It seems that the coexistence of the three alleles could be a major risk of developing CD and that they may play a role in phenotype determination.

Haplotype frequency analysis of the *CARD15/NOD2* gene showed that the haplotype with the Arg702Trp confers the single highest genetic risk for disease. This result is in agreement with previous studies in a Spanish population [29]. However, this mutation was not associated to CD in Galician, Finnish or Scottish populations [30]. The risk for CD was much greater in the presence of more than one of the studied *CARD15/NOD2* variants.

The presented data suggest a need for further work and larger studies to determine other genetic and environmental factors influencing CD susceptibility and behavior in the Saudi population.

## Supplementary Information



## Figures and Tables

**Table 1:** Distribution of Chron’s disease (CD) patients according to demographic data.

	No.	%
**Sex**		
Male	31	67.4
Female	15	32.6
**Age**		
Range	18.0–70.0	
Mean ± SD	30.43 ± 10.20	
**Smoking**		
No	39	84.8
Yes	7	15.2
**Family IBD Hx**		
No	37	80.4
Yes	9	19.6
**Complication**		
No not for surgery	23	50.0
Yes for surgery	23	50.0
**Phenotype**		
Fistulizing	22	47.8
Fibrostenotic	7	15.2
Inflammatory	16	34.8
Fistulizing & Fibrostenotic	1	2.2
**Presenting symptoms**		
Abdominal pain	38	86.4
Anemia	7	15.9
Arthritis	6	13.6
Bleeding per rectum	19	43.2
Diarrhea	33	75.0
Eye disease	3	6.8
Fever	10	22.7
Perianal Disease	16	36.4
Skin lesions	4	9.1
Vomiting	6	13.6
Weight loss	24	54.5
**Medications**		
5-ASA	23	51.1
Adalimumab	3	6.7
Budesonide	3	6.7
Imuran	35	77.8
Infliximab	28	62.2
Steroid	12	26.7
**Disease extent**		
Ileal	10	21.7
Ileocolonic	27	58.7
Colonic	9	19.6

**Table 2:** *CARD15/NOD2* mutant allele frequencies in Saudi Crohn’s disease (CD) patients and controls.

	Homozygous wild-type	Homozygous mutant	*P*	Heterozygous mutant	*P*	Or (95% ci) mutant	Or (95% ci) hetero
**Leu1007fsinsC**							
Patients	7 (15.2%)	3 (6.5%)	0.012 *	36 (78.3%)	<0.0001 *	5.29 (2.71–10.29)	7.71 (2.87–20.71)
Controls	30 (60.0%)	0 (0.0%)		20 (20.0%)			
**Arg702Trp**							
Patients	8 (17.4%)	10 (21.7%)	<0.0001 *	28 (60.6%)	<0.0001 *	21.87 (3.99–119.9)	9.42 (3.43–25.9)
Controls	35 (70.0%)	2 (4.0%)		13 (26.0%)			
**Gly908Arg**							
Patients	5 (10.9%)	3 (6.5%)	0.015 *	38 (82.6%)	<0.0001 *	21.0 (1.81–243.2)	19.0 (6.20–58.21)
Controls	35 (70.0%)	1 (2.0%)		14 (28.0%)			

* Statistically significant at *P* ≤ 0.05.

**Table 3:** Allele and genotype frequencies for toll-like receptor-4 (TLR4) Thr399Ile and the CD14 −159C/T polymorphism in Saudi CD patients.

	Homozygous wild-type	Homozygous mutant	*P*	Heterozygous	*P*	Or (95% ci) mutant	Or (95% ci) hetero
**TLR4 Thr399Ile**							
Patients	24 (52.2%)	4 (8.7%)	0.495	18 (39.1%)	0.226	1.33 (0.30–5.88)	1.71 (0.71–4.12)
Control	32 (64.0%)	4 (8.0%)		14 (28.0)			
**CD14 −159C/T gene**							
Patients	13 (28.3%)	7 (15.2%)	0.075	26 (56.5%)	0.002 *	3.23 (0.86–12.09)	4.0 (1.61–9.93)
Control	30 (60.0%)	5 (10.0%)		15 (30.0%)			

* Statistically significant at *P* ≤ 0.05.

**Table 4:** Comparison between Leu1007fsinsC, Arg702Trp, Gly908Arg, TLR4 Thr399Ile and CD14 −159C/T.

	Leu1007fsinsC		Arg702Trp		Gly908Arg		TLR4 Thr399Ile		CD14 −159C/T	

	No.	%	No.	%	No.	%	No.	%	No.	%
Wild	7	15.2	8	17.4	5	10.9	24	52.2	13	28.3
Mutant	3	6.5	10	21.7	3	77	4	8.7	7	15.2
Hetero	36	78.3	28	60.9	38	26	18	39.1	26	56.5
***P*_1_**			*P* = 0.089		*P* = 0.824		*P* < 0.001^*^		*P* = 0.082	
***P*_2_**					*P* = 0.0504		*P* = 0.002^*^		*P* = 0.408	
***P*_3_**							*P* = 0.025^*^		*P* < 0.001^*^	
***P*_4_**									*P* = 0.063	

*P*_1_: *P* value between Leu1007fsinsC and each other mutation; *P*_2_: *P* value between Arg702Trp and each other mutation; *P*_3_: *P* value between Gly908Arg and each other mutation; *P*_4_: *P* value between TLR4 Thr399Ile and CD14 −159C/T.

**Table 5:** Comparisons between the two groups studied according to combinations of mutations.

	Patients		Control	
	
	No.	%	No.	%
None of the mutant alleles	0	0.0	7	14.0
*CARD15/NOD2*	16	34.8	20	40.0
TLR4 Thr399Ile **only**	0	0.0	3	6.0
CD14 −159C/T **gene**	1	2.2	1	2.0
*CARD15/NOD2*/TLR4 Thr399Ile	4	8.7	5	10.0
*CARD15/NOD2***/**CD14 −159C/T	11	23.9	8	16.0
TLR4 Thr399Ile/CD14 −159C/T *CARD15/NOD2*/TLR4	0	0.0	2	4.0
Thr399Ile/CD14 −159C/T **gene**	14	30.4	4	8.0

**Table 6:** Haplotypes analysis of *CARD15/NOD2* polymorphism in Crohn’s disease patients and controls.

Leu1007fsinsC	Arg702Trp	Gly908Arg	Number in patients/47	Number in control/50	*P-value	* Odds ratio	95% confidence intervals
1	1	1	28 (59.57%)	14 (28%)	0.002	3.789	1.62–8.86
2	1	1	30 (63.82%)	23 (46%)	0.078	2.072	0.92–4.68
1	1	2	30 (63.8%)	36 (72%)	0.388	0.686	0.29–1.62
1	2	1	30 (63.8%)	14 (28%)	*P* < 0.0001	4.538	1.93–10.69
1	2	2	32 (68.08%)	12 (24%)	*P* < 0.0001	6.756	2.77–16.49
2	1	2	29 (61.70%)	17 (34%)	0.006	3.127	1.36–7.17
2	2	1	32 (68.08%)	3 (6%)	*P* < 0.0001	33.422	8.94–124.92
2	2	2	31 (65.95%)	2 (4%)	*P* < 0.0001	46.50	9.99–216.42

1, wild-type; 2, mutant;

* Odds ratio of disease status of each haplotype (1-1-1 reference haplotype);

* *P*-value by chi square test.

**Table 7:** Primers and polymerase chain reaction (PCR) conditions used for genotyping *CARD15/NOD2*, TLR4 and CD14 genes.

Gene mutation	Primer name	Primer Sequence 5′-3′	Cycling	Expected product
*CARD15/NOD2* cytosine insertion mutation	Leu1007fsinsCW TF,	CAGAAGCCCTCCTGCAGGCCCT	35 cycles of: 94 °C for 45 s, 65 °C for 40 s, 72 °C for 30 s	+ve for wild
	Leu1007fsinsCR (common reverse)	TCTTCAACCACATCCCCATT		
	
	Leu1007fsinsCMUTF	CAGAAGCCCTCCTGCAGGCCCCT		+ve for mutant
	Leu1007fsinsCR(common reverse)	TCTTCAACCACATCCCCATT		

*CARD15/NOD2* Missense mutation Arg702Trp	R702WWTF	ATCTGAGAAGGCCCTGCTCC	35 cycles of: 94 °C for 45 s, 53 °C for 40 s, 72 °C for 30 s	+ve for wild
	R702WR, (common reverse)	CCCACACTTAGCCTTGATG		
	
	R702WMUTF	ATCTGAGAAGGCCCTGCTCT		+ve for mutant
	R702WR,(common reverse)	CCCACACTTAGCCTTGATG		

*CARD15/NOD2* Gly908Arg	Gly908Arg F	CCCAGCTCCTCCCTCTTC	35 cycles of: 94 °C for 45 s, 53 °C for 40 s, 72 °C for 30 s	For wild 380 bp fragment with *Hha*I digest
	Gly908Arg R	AAGTCTGTAATGTAAAGCCAC		

TLR4 Thr399Ile	Thr399Ile FW	GGTTGCTGTTCTCAAAGTGATTTTGGGAGAA	35 cycles of: 95 °C for 30 s, 55 °C for 30 s, 72 °C for 30 s	For wild 377 bp With *Hinf*I restriction For mutant 223 bp with *Hinf*I restriction
	Thr399Ile R	CCTGAAGACTGGAGAGTGAGTTAAATGCT		

CD14 −159C/T	CDP-1	TTGGTGCCAACAGATGAGGTTCAC	35 cycles of: 92 °C for 40 s, 62 °C for 35 s, 72 °C for 50 s.	For wild 204, 201 and 156 bp. With *Hae*III digest For mutant 360 and 201 bp. With *Hae*III digest
	CDP-2	TTCTTTCCTACACAGCGGCACCC		
