# Supplementary material for: CARD15/NOD2, CD14 and Toll-like 4 Receptor Gene Polymorphisms in Saudi Patients with Crohn’s Disease
Source: Int J Mol Sci. 2012 Apr 2;13(4):4268–80. doi: 10.3390/ijms13044268 (PMC3344213; doi:10.3390/ijms13044268)
Supplement: Supplementary file 1 [file ijms-13-04268-s001.pdf]

Supplementary Information

1. Analysis of the Association of the Three Variants of *CARD15/NOD2,* TLR4 (Thr399Il) and CD14 −159C/T with Family History

Table S1. *CARD15/NOD2* Leu1007fsinsC frequency with family history.

			FH		Total
			+	−	
Leu1007fsinsC	W	Count	1	6	7
		% Leu1007fsinsC	14.3%	85.7%	100.0%
		% within FH	11.1%	17.6%	16.3%
	M	Count	8	28	36
		% Leu1007fsinsC	22.2%	77.8%	100.0%
		% within FH	88.9%	82.4%	83.7%
Total		Count	9	34	43
		% Leu1007fsinsC	20.9%	79.1%	100.0%
		% within FH	100.0%	100.0%	100.0%

Table S2. Analysis of association of *CARD15/NOD2* Leu1007fsinsC polymorphism with family history Using Chi-Square Tests.

	Value	df	Asymp. Sig. (2-sided)	Exact Sig. (2-sided)	Exact Sig. (1-sided)
Pearson Chi-Square	0.223 [a]	1	0.637		
Continuity Correction [b]	0.000	1	1.000		
Likelihood Ratio	0.240	1	0.624		
Fisher’s Exact Test				1.000	0.543
Linear-by-Linear Association	0.218	1	0.641		
N of Valid Cases	43				

[a] 1 cells (25.0%) have expected count less than 5. The minimum expected count is 1.47; [b] Computed only for a 2 × 2 table.

Table S3. *CARD15/NOD2* Arg702Trp frequency with family history.

			FH		Total
			+	−	
Misense Arg702Trp	W	Count	1	5	6
		% Arg702Trp	16.7%	83.3%	100.0%
		% within FH	11.1%	14.7%	14.0%
	M	Count	8	29	37
		% Arg702Trp	21.6%	78.4%	100.0%
		% within FH	88.9%	85.3%	86.0%
Total		Count	9	34	43
		% Arg702Trp	20.9%	79.1%	100.0%
		% within FH	100.0%	100.0%	100.0%

Table S4. Analysis of association of *CARD15/NOD2* Arg702Trp polymorphism with family history Using Chi-Square Tests.

	Value	df	Asymp. Sig. (2-sided)	Exact Sig. (2-sided)	Exact Sig. (1-sided)
Pearson Chi-Square	0.077 [a]	1	0.782		
Continuity Correction [b]	0.000	1	1.000		
Likelihood Ratio	0.080	1	0.777		
Fisher's Exact Test				1.000	0.631
Linear-by-Linear Association	0.075	1	0.784		
N of Valid Cases	43				

[a] 2 cells (50.0%) have expected count less than 5. The minimum expected count is 1.26; [b] Computed only for a 2 × 2 table.

Table S5. *CARD15/NOD2* G908R frequency with family history.

			FH		Total
			+	−	
Missense Mutation (hHa)	W	Count	0	5	5
		% G908R	0.0%	100.0%	100.0%
		% within FH	0.0%	14.7%	11.6%
	M	Count	9	29	38
		% G908R	23.7%	76.3%	100.0%
		% within FH	100.0%	85.3%	88.4%
Total		Count	9	34	43
		% G908R	20.9%	79.1%	100.0%
		% within FH	100.0%	100.0%	100.0%

Table S6. Analysis of association of *CARD15/NOD2* G908R polymorphism with family history using Chi-Square Tests.

	Value	df	Asymp. Sig. (2-sided)	Exact Sig. (2-sided)	Exact Sig. (1-sided)
Pearson Chi-Square	1.498[a]	1	0.221		
Continuity Correction [b]	0.408	1	0.523		
Likelihood Ratio	2.517	1	0.113		
Fisher's Exact Test				0.566	0.289
Linear-by-Linear Association	1.463	1	0.226		
N of Valid Cases	43				

[a] 2 cells (50.0%) have expected count less than 5. The minimum expected count is 1.05; [b] Computed only for a 2 × 2 table.

Table S7. TLR4 Thr399Ile frequency with family history.

			FH		
			+	−	**Total**
Hinf 1	W	Count	1	20	21
		% TLR4 Thr399Ile	4.8%	95.2%	100.0%
		% within FH	11.1%	58.8%	48.8%
	M	Count	8	14	22
		% TLR4 Thr399Ile	36.4%	63.6%	100.0%
		% within FH	88.9%	41.2%	51.2%
Total		Count	9	34	43
		% TLR4 Thr399Ile	20.9%	79.1%	100.0%
		% within FH	100.0%	100.0%	100.0%

Table S8. Analysis of association of TLR4 Thr399Ile polymorphism with family history using Chi-Square Tests.

	Value	df	Asymp. Sig. (2-sided)	Exact Sig. (2-sided)	Exact Sig. (1-sided)
Pearson Chi-Square	6.484 [a]	1	0.011		
Continuity Correction [b]	4.715	1	0.030		
Likelihood Ratio	7.239	1	0.007		
Fisher's Exact Test				0.021	0.013
Linear-by-Linear Association	6.333	1	0.012		
N of Valid Cases	43				

[a] 2 cells (50.0%) have expected count less than 5. The minimum expected count is 4.40; [b] Computed only for a 2 × 2 table.

Table S9. CD14 −159(C/T) frequency with family history.

			FH		
			+	−	**Total**
Hae	W	Count	3	9	12
		% −159(C/T)	25.0%	75.0%	100.0%
		% within FH	33.3%	26.5%	27.9%
	M	Count	6	25	31
		% −159(C/T)	19.4%	80.6%	100.0%
		% within FH	66.7%	73.5%	72.1%
Total		Count	9	34	43
		% −159(C/T)	20.9%	79.1%	100.0%
		% within FH	100.0%	100.0%	100.0%

Table S10. Analysis of association of CD14-159(C/T) polymorphism with family history using Chi-Square Tests.

	Value	df	Asymp. Sig. (2-sided)	Exact Sig. (2-sided)	Exact Sig. (1-sided)
Pearson Chi-Square	0.167 [a]	1	0.683		
Continuity Correction [b]	0.000	1	1.000		
Likelihood Ratio	0.162	1	0.687		
Fisher's Exact Test				0.692	0.489
Linear-by-Linear Association	0.163	1	0.687		
N of Valid Cases	43				

[a] 1 cells (25.0%) have expected count less than 5. The minimum expected count is 2.51; [b] Computed only for a 2 × 2 table.

2. Analysis of the Association of the Three Variants of *CARD15/NOD2,* TLR4 (Thr399Il) and CD14 −159C/T with Sex

Table S11. *CARD15/NOD2* Leu1007fsinsC frequency with sex.

			Sex		
			**Male**	**Female**	**Total**
Leu1007fsinsC	W	Count	2	4	6
		% Leu1007fsinsC	33.3%	66.7%	100.0%
		% within Sex	7.1%	26.7%	14.0%
	M	Count	26	11	37
		% Leu1007fsinsC	70.3%	29.7%	100.0%
		% within Sex	92.9%	73.3%	86.0%
Total		Count	28	15	43
		% Leu1007fsinsC	65.1%	34.9%	100.0%
		% within Sex	100.0%	100.0%	100.0%

Table S12. Analysis of association of *CARD15/NOD2* Leu1007fsinsC polymorphism with Sex using Chi-Square Tests.

	Value	df	Asymp. Sig. (2-sided)	Exact Sig. (2-sided)	Exact Sig. (1-sided)
Pearson Chi-Square	3.101 [a]	1	0.078		
Continuity Correction [b]	1.688	1	0.194		
Likelihood Ratio	2.947	1	0.086		
Fisher's Exact Test				0.161	0.099
Linear-by-Linear Association	3.029	1	0.082		
N of Valid Cases	43				

[a] 2 cells (50.0%) have expected count less than 5. The minimum expected count is 2.09; [b] Computed only for a 2 × 2 table.

Table S13. *CARD15/NOD2* Arg702Trp frequency with sex.

			Sex		
			**Male**	**Female**	**Total**
Arg702Trp	W	Count	7	0	7
		% Arg702Trp	100.0%	0.0%	100.0%
		% within Sex	25.0%	0.0%	16.3%
	M	Count	21	15	36
		% Arg702Trp	58.3%	41.7%	100.0%
		% within Sex	75.0%	100.0%	83.7%
Total		Count	28	15	43
		% Arg702Trp	65.1%	34.9%	100.0%
		% within Sex	100.0%	100.0%	100.0%

Table S14. Analysis of association of *CARD15/NOD2* Arg702Trp polymorphism with sex using Chi-Square Tests.

	**Value**	**df**	**Asymp. Sig. (2-sided)**	**Exact Sig. (2-sided)**	**Exact Sig. (1-sided)**
Pearson Chi-Square	4.479 [a]	1	0.034		
Continuity Correction [b]	2.833	1	0.092		
Likelihood Ratio	6.716	1	0.010		
Fisher's Exact Test				0.077	0.037
Linear-by-Linear Association	4.375	1	0.036		
N of Valid Cases	43				

[a] 2 cells (50.0%) have expected count less than 5. The minimum expected count is 2.44; [b] Computed only for a 2 × 2 table.

Table S15. *CARD15/NOD2* G908R frequency with sex.

			Sex		
			**Male**	**Female**	**Total**
G908R	W	Count	3	2	5
		% G908R	60.0%	40.0%	100.0%
		% within Sex	10.7%	13.3%	11.6%
	M	Count	25	13	38
		% G908R	65.8%	34.2%	100.0%
		% within Sex	89.3%	86.7%	88.4%
Total		Count	28	15	43
		% G908R	65.1%	34.9%	100.0%
		% within Sex	100.0%	100.0%	100.0%

Table S16. Analysis of association of *CARD15/NOD2* G908R polymorphism with sex using Chi-Square Tests.

	Value	df	Asymp. Sig. (2-sided)	Exact Sig. (2-sided)	Exact Sig. (1-sided)
Pearson Chi-Square	0.065 [a]	1	0.798		
Continuity Correction [b]	0.000	1	1.000		
Likelihood Ratio	0.064	1	0.800		
Fisher's Exact Test				1.000	0.579
Linear-by-Linear Association	0.064	1	0.801		
N of Valid Cases	43				

[a] 2 cells (50.0%) have expected count less than 5. The minimum expected count is 1.74; [b] Computed only for a 2 × 2 table.

Table S17. TLR4 Thr399Ile frequency with sex.

			Sex		
			**Male**	**Female**	**Total**
Thr399Ile	W	Count	14	8	22
		% Thr399Ile	63.6%	36.4%	100.0%
		% within Sex	50.0%	53.3%	51.2%
	M	Count	14	7	21
		% Thr399Ile	66.7%	33.3%	100.0%
		% within Sex	50.0%	46.7%	48.8%
Total		Count	28	15	43
		% within Hinf 1	65.1%	34.9%	100.0%
		% within Sex	100.0%	100.0%	100.0%

Table S18. Analysis of association of TLR4 Thr399Ile polymorphism with sex using Chi-Square Tests.

	Value	df	Asymp. Sig. (2-sided)	Exact Sig. (2-sided)	Exact Sig. (1-sided)
Pearson Chi-Square	0.043 [a]	1	0.835		
Continuity Correction [b]	0.000	1	1.000		
Likelihood Ratio	0.043	1	0.835		
Fisher's Exact Test				1.000	0.545
Linear-by-Linear Association	0.042	1	0.837		
N of Valid Cases	43				

[a] 0 cells (.0%) have expected count less than 5. The minimum expected count is 7.33; [b] Computed only for for a 2 × 2 table.

Table S19. CD14 −159(C/T) frequency with sex.

			Sex		
			**Male**	**Female**	**Total**
-159(C/T)	W	Count	6	6	12
		% −159(C/T)	50.0%	50.0%	100.0%
		% within Sex	21.4%	40.0%	27.9%
	M	Count	22	9	31
		% −159(C/T)	71.0%	29.0%	100.0%
		% within Sex	78.6%	60.0%	72.1%
Total		Count	28	15	43
		% −159(C/T)	65.1%	34.9%	100.0%
		% within Sex	100.0%	100.0%	100.0%

Table S20. Analysis of association of CD14 −159(C/T) polymorphism with sex using Chi-Square Tests.

	**Value**	**df**	**Asymp. Sig. (2-sided)**	**Exact Sig. (2-sided)**	**Exact Sig. (1-sided)**
Pearson Chi-Square	1.674 [a]	1	0.196		
Continuity Correction [b]	0.879	1	0.349		
Likelihood Ratio	1.631	1	0.202		
Fisher's Exact Test				0.287	0.174
Linear-by-Linear Association	1.635	1	0.201		
N of Valid Cases	43				

[a] 1 cells (25.0%) have expected count less than 5. The minimum expected count is 4.19; [b] Computed only for a 2 × 2 table.

3. Analysis of the Association of the Three Variants of *CARD15/NOD2*, TLR4 (Thr399Il) and CD14–159C/T with Pheotypes

Table S21. *CARD15/NOD2* Leu1007fsinsC frequency with phenotypes.

				Phenotype		
			**Inflammatory**	**Fistulizing**	**Fibrostenotic**	**Total**
Leu1007fsinsC	W	Count	3	2	0	5
		% Leu1007fsinsC	60.0%	40.0%	0.0%	100.0%
		% within Phenotype	21.4%	10.0%	0.0%	12.2%
	M	Count	11	18	7	36
		% Leu1007fsinsC	30.6%	50.0%	19.4%	100.0%
		% within Phenotype	78.6%	90.0%	100.0%	87.8%
Total		Count	14	20	7	41
		% Leu1007fsinsC	34.1%	48.8%	17.1%	100.0%
		% within Phenotype	100.0%	100.0%	100.0%	100.0%

Table S22. Analysis of association of *CARD15/NOD2* Leu1007fsinsC polymorphism with phenotypes using Chi-Square Tests.

	Value	df	Asymp. Sig. (2-sided)
Pearson Chi-Square	2.177 [a]	2	0.337
Likelihood Ratio	2.854	2	0.240
Linear-by-Linear Association	2.119	1	0.145
N of Valid Cases	41		

[a] 3 cells (50.0%) have expected count less than 5. The minimum expected count is 0.85.

Table S23. *CARD15/NOD2* Arg702Trp frequency with phenotypes.

			Phenotype			
			**Inflammatory**	**Fistulizing**	**Fibrostenotic**	**Total**
Arg702Trp	W	Count	1	4	1	6
		% Arg702Trp	16.7%	66.7%	16.7%	100.0%
		% within Phenotype	7.1%	20.0%	14.3%	14.6%
	M	Count	13	16	6	35
		% Arg702Trp	37.1%	45.7%	17.1%	100.0%
		% within Phenotype	92.9%	80.0%	85.7%	85.4%
Total		Count	14	20	7	41
		% Arg702Trp	34.1%	48.8%	17.1%	100.0%
		% within Phenotype	100.0%	100.0%	100.0%	100.0%

Table S24. Analysis of association of *CARD15/NOD2* Arg702Trp polymorphism with phenotypes using Chi-Square Tests.

	Value	df	Asymp. Sig. (2-sided)
Pearson Chi-Square	1.091 [a]	2	0.580
Likelihood Ratio	1.175	2	0.556
Linear-by-Linear Association	0.414	1	0.520
N of Valid Cases	41		

[a] 3 cells (50.0%) have expected count less than 5. The minimum expected count is 1.02.

Table S25. *CARD15/NOD2* G908R frequency with phenotypes.

			Phenotype			
			**Inflammatory**	**Fistulizing**	**Fibrostenotic**	**Total**
G908R	W	Count	3	1	1	5
		% G908R	60.0%	20.0%	20.0%	100.0%
		% within Phenotype	21.4%	5.0%	14.3%	12.2%
	M	Count	11	19	6	36
		% G908R	30.6%	52.8%	16.7%	100.0%
		% within Phenotype	78.6%	95.0%	85.7%	87.8%
Total		Count	14	20	7	41
		% G908R	34.1%	48.8%	17.1%	100.0%
		% within Phenotype	100.0%	100.0%	100.0%	100.0%

Table S26. Analysis of association of *CARD15/NOD2* G908R polymorphism phenotypes using Chi-Square Tests.

	Value	df	Asymp. Sig. (2-sided)
Pearson Chi-Square	2.110[a]	2	0.348
Likelihood Ratio	2.175	2	0.337
Linear-by-Linear Association	0.605	1	0.437
N of Valid Cases	41		

[a] 3 cells (50.0%) have expected count less than 5. The minimum expected count is 0.85.

Table S27. TLR4 Thr399Ile frequency with phenotypes.

			Phenotype			
			Inflammatory	Fistulizing	Fibrostenotic	Total
Thr399Ile	W	Count	8	9	3	20
		% Thr399Ile	40.0%	45.0%	15.0%	100.0%
		% within Phenotype	57.1%	45.0%	42.9%	48.8%
	M	Count	6	11	4	21
		% Thr399Ile	28.6%	52.4%	19.0%	100.0%
		% within Phenotype	42.9%	55.0%	57.1%	51.2%
Total		Count	14	20	7	41
		% Thr399Ile	34.1%	48.8%	17.1%	100.0%
		% within Phenotype	100.0%	100.0%	100.0%	100.0%

Table S28. Analysis of association of TLR4 Thr399Ile polymorphism with phenotypes Using Chi-Square Tests.

	Value	df	Asymp. Sig. (2-sided)
Pearson Chi-Square	0.605[a]	2	0.739
Likelihood Ratio	0.606	2	0.739
Linear-by-Linear Association	0.496	1	0.481
N of Valid Cases	41		

[a] 2 cells (33.3%) have expected count less than 5. The minimum expected count is 3.41.

Table S29. CD14 −159(C/T) frequency with phenotypes.

			Phenotype			
			Inflammatory	Fistulizing	Fibrostenotic	Total
−159(C/T)	W	Count	6	4	2	12
		% −159(C/T)	50.0%	33.3%	16.7%	100.0%
		% within Phenotype	42.9%	20.0%	28.6%	29.3%
	M	Count	8	16	5	29
		% −159(C/T)	27.6%	55.2%	17.2%	100.0%
		% within Phenotype	57.1%	80.0%	71.4%	70.7%
Total		Count	14	20	7	41
		% −159(C/T)	34.1%	48.8%	17.1%	100.0%
		% within Phenotype	100.0%	100.0%	100.0%	100.0%

Table S30. Analysis of association of CD14 −159(C/T) polymorphism with phenotypes Using Chi-Square Tests.

	Value	df	Asymp. Sig. (2-sided)
Pearson Chi-Square	2.080 [a]	2	0.353
Likelihood Ratio	2.059	2	0.357
Linear-by-Linear Association	0.906	1	0.341
N of Valid Cases	41		

[a] 3 cells (50.0%) have expected count less than 5. The minimum expected count is 2.05.

4. Analysis of the Association of the Three Variants of *CARD15/NOD2* , TLR4 (Thr399Il) and CD14–159C/T with Family History

Table S31. *CARD15/NOD2* Leu1007fsinsC frequency with smoking.

			Smoking		
			Yes	No	Total
Leu1007fsinsC	W	Count	0	6	6
		% Leu1007fsinsC	0.0%	100.0%	100.0%
		% within Smoking	0.0%	17.1%	15.4%
	M	Count	4	29	33
		% Leu1007fsinsC	12.1%	87.9%	100.0%
		% within Smoking	100.0%	82.9%	84.6%
Total		Count	4	35	39
		% Leu1007fsinsC	10.3%	89.7%	100.0%
		% within Smoking	100.0%	100.0%	100.0%

Table S32. Analysis of association of *CARD15/NOD2* Leu1007fsinsC polymorphism with family smoking Using Chi-Square Tests.

	Value	df	Asymp. Sig. (2-sided)	Exact Sig. (2-sided)	Exact Sig. (1-sided)
Pearson Chi-Square	0.810 [a]	1	0.368		
Continuity Correction [b]	0.028	1	0.866		
Likelihood Ratio	1.417	1	0.234		
Fisher's Exact Test				1.000	0.498
Linear-by-Linear Association	0.790	1	0.374		
N of Valid Cases	39				

[a] 2 cells (50.0%) have expected count less than 5. The minimum expected count is 0.62; [b] Computed only for a 2 × 2 table.

Table S33. *CARD15/NOD2* Arg702Trp frequency with smoking.

			Smoking		
			**Yes**	**No**	**Total**
Arg702Trp	W	Count	2	4	6
		% Arg702Trp	33.3%	66.7%	100.0%
		% within Smoking	50.0%	11.4%	15.4%
	M	Count	2	31	33
		% Arg702Trp	6.1%	93.9%	100.0%
		% within Smoking	50.0%	88.6%	84.6%
Total		Count	4	35	39
		% Arg702Trp	10.3%	89.7%	100.0%
		% within Smoking	100.0%	100.0%	100.0%

Table S34. Analysis of association of *CARD15/NOD2* Arg702Trp polymorphism with smoking using Chi-Square Tests.

	**Value**	**df**	**Asymp. Sig. (2-sided)**	**Exact Sig. (2-sided)**	**Exact Sig. (1-sided)**
Pearson Chi-Square	4.103 [a]	1	0.043		
Continuity Correction [b]	1.675	1	0.196		
Likelihood Ratio	3.065	1	0.080		
Fisher's Exact Test				0.104	0.104
Linear-by-Linear Association	3.997	1	0.046		
N of Valid Cases	39				

[a] 2 cells (50.0%) have expected count less than 5. The minimum expected count is 0.62; [b] Computed only for a 2 × 2 table.

Table S35. *CARD15/NOD2* G908R frequency with smoking.

			Smoking		
			**Yes**	**No**	**Total**
G908R	W	Count	1	2	3
		% G908R	33.3%	66.7%	100.0%
		% within Smoking	25.0%	5.7%	7.7%
	M	Count	3	33	36
		% G908R (hHa)	8.3%	91.7%	100.0%
		% within Smoking	75.0%	94.3%	92.3%
Total		Count	4	35	39
		% G908R	10.3%	89.7%	100.0%
		% within Smoking	100.0%	100.0%	100.0%

Table S36. Analysis of association of *CARD15/NOD2* G908R polymorphism with smoking using Chi-Square Tests.

	Value	df	Asymp. Sig. (2-sided)	Exact Sig. (2-sided)	Exact Sig. (1-sided)
Pearson Chi-Square	1.880 [a]	1	0.170		
Continuity Correction [b]	0.145	1	0.703		
Likelihood Ratio	1.322	1	0.250		
Fisher's Exact Test				0.284	0.284
Linear-by-Linear Association	1.832	1	0.176		
N of Valid Cases	39				

[a] 3 cells (75.0%) have expected count less than 5. The minimum expected count is 0.31; [b] Computed only for a 2 × 2 table.

Table S37. TLR4 Thr399Ile frequency with smoking.

			Smoking		
			Yes	No	Total
Thr399Ile	W	Count	1	17	18
		% Thr399Ile	5.6%	94.4%	100.0%
		% within Smoking	25.0%	48.6%	46.2%
	M	Count	3	18	21
		% Thr399Ile	14.3%	85.7%	100.0%
		% within Smoking	75.0%	51.4%	53.8%
Total		Count	4	35	39
		% Thr399Ile	10.3%	89.7%	100.0%
		% within Smoking	100.0%	100.0%	100.0%

Table S38. Analysis of association of TLR4 Thr399Ile polymorphism with smoking using Chi-Square Tests.

	Value	df	Asymp. Sig. (2-sided)	Exact Sig. (2-sided)	Exact Sig. (1-sided)
Pearson Chi-Square	0.803 [a]	1	0.370		
Continuity Correction [b]	0.134	1	0.714		
Likelihood Ratio	0.844	1	0.358		
Fisher's Exact Test				0.609	0.364
Linear-by-Linear Association	0.782	1	0.377		
N of Valid Cases	0.39				

[a] 2 cells (50.0%) have expected count less than 5. The minimum expected count is 1.85; [b] Computed only for a 2 × 2 table.

Table S39. CD14 −159(C/T) frequency with smoking.

			Smoking		
			**Yes**	**No**	**Total**
CD14-159(C/T)	W	Count	1	9	10
		% −159(C/T)	10.0%	90.0%	100.0%
		% within Smoking	25.0%	25.7%	25.6%
	M	Count	3	26	29
		% −159(C/T)	10.3%	89.7%	100.0%
		% within Smoking	75.0%	74.3%	74.4%
Total		Count	4	35	39
		% −159(C/T)	10.3%	89.7%	100.0%
		% within Smoking	100.0%	100.0%	100.0%

Table S40. Analysis of association of *CARD15/NOD2* Leu1007fsinsC polymorphism with smoking 0.35 Using Chi-Square Tests.

	**Value**	**df**	**Asymp. Sig. (2-sided)**	**Exact Sig. (2-sided)**	**Exact Sig. (1-sided)**
Pearson Chi-Square	0.001 [a]	1	0.975		
Continuity Correction [b]	0.000	1	1.000		
Likelihood Ratio	0.001	1	0.975		
Fisher's Exact Test				1.000	0.733
Linear-by-Linear Association	0.001	1	0.976		
N of Valid Cases	39				

[a] 2 cells (50.0%) have expected count less than 5. The minimum expected count is 1.03; [b] Computed only for a 2 × 2 table.

© 2012 by the authors; licensee MDPI, Basel, Switzerland. This article is an open access article distributed under the terms and conditions of the Creative Commons Attribution license (http://creativecommons.org/licenses/by/3.0/).
